# Tracing the STING exocytosis pathway during herpes viruses infection

**DOI:** 10.1128/mbio.00373-24

**Published:** 2024-03-12

**Authors:** Christos Dogrammatzis, Rabina Saud, Hope Waisner, Sarah Lasnier, Sreenath Muraleedharan Suma, Brandon Grieshaber, Maria Kalamvoki

**Affiliations:** 1 Department of Microbiology, Molecular Genetics and Immunology, University of Kansas Medical Center, Kansas City, Kansas, USA; National Institutes of Health, Bethesda, Maryland, USA; Washington State University, Pullman, Washington, USA

**Keywords:** extracellular vesicles, tetraspanins, CD63, HSV-1, STING, STIM1 innate immunity

## Abstract

The STimulator of INterferon Genes (STING) constitutes a major DNA-sensing pathway that restricts HSV-1 infection in different models by activating type I interferon and pro-inflammatory responses. To counteract STING, HSV-1 has evolved numerous strategies including mechanisms to interfere with its oligomerization, post-translational modifications, and downstream signaling. Previously, we demonstrated that STING is packaged in extracellular vesicles (EVs) produced from HSV-1-infected cells. These EVs activated antiviral responses in uninfected recipient cells and suppressed a subsequent HSV-1 infection in a STING-dependent manner. Here, we provide information on the packaging of STING in EVs and its exocytosis. We found that STING exocytosis did not occur in CD63 knockdown cells supporting that STING follows the CD63 exocytosis pathway. Consistently, we found that STING co-localized with CD63 in cytoplasmic globular structures and exosomal STING and CD63 co-fractionated. Both golgicide A and brefeldin A prevented STING exocytosis during HSV-1 infection suggesting that STING trafficking through the Golgi is required. A STING ligand was insufficient for STING exocytosis, and downstream signaling through TBK1 was not required. However, STING palmitoylation and tethering to the ER by STIM1 were required for STING exocytosis. Finally, we found that HSV-1 replication/late gene expression triggered CD63 exocytosis that was required for STING exocytosis. Surprisingly, HSV-2 strain G did not trigger CD63 or STING exocytosis as opposed to VZV and HCMV. Also, EVs from HSV-1(F)- and HSV-2(G)-infected cells displayed differences in their ability to restrict these viruses. Overall, STING exocytosis is induced by certain viruses and shapes the microenvironment of infection.

**IMPORTANCE:**

Extracellular vesicles (EVs) are released by all types of cells as they constitute a major mechanism of intercellular communication. The packaging of specific cargo in EVs and the pathway of exocytosis are not fully understood. STING is a sensor of a broad spectrum of pathogens and a key component of innate immunity. STING exocytosis during HSV-1 infection has been an intriguing observation, raising questions of whether this is a virus-induced process, the purpose it serves, and whether it is observed after infection with other viruses. Here, we have provided insights into the pathway of STING exocytosis and determined factors involved. STING exocytosis is a virus-induced process and not a response of the host to the infection. Besides HSV-1, other herpes viruses triggered STING exocytosis, but HSV-2(G) did not. HSV-1 EVs displayed different restriction capabilities compared with HSV-2(G) EVs. Overall, STING exocytosis is triggered by viruses to shape the microenvironment of infection.

## INTRODUCTION

Since its discovery in 2008, STING has attracted the attention of different fields, particularly infectious diseases, immunology, and cancer biology due to its roles in mounting antimicrobial or antitumor immunity and autoimmune disorders (1–7). STING is a key component of a major double-stranded DNA-sensing pathway that leads to activation of type I interferon and pro-inflammatory responses (8–11). Double-stranded DNA (dsDNA) that is sensed by STING usually originates from pathogens invading the host, or self-DNA released in the cytoplasm of cells, for example, due to aging, trauma, or from cancerous cells with damaged mitochondria and nuclei (12–15). Aberrant self-DNA in the cytoplasm that can activate STING leads to exacerbation of autoimmune disorders and other inflammatory syndromes (11).

Human STING is a 379 aa protein that resides in the endoplasmic reticulum (ER) and consists of four transmembrane domains at the N-terminus, a dimerization domain within the ligand-binding domain that allows for the formation of a “pocket” for the binding of ligands, and a C-terminal tail in the cytosol for signaling (4, 9, 10, 16, 17). STING can sense bacterial secondary messengers such as the cyclin di-GMP (CDG), cyclin di-AMP, and dsDNA oligonucleotides. STING point mutations have indicated that cyclic dinucleotide sensing and DNA sensing can be uncoupled, suggesting that the two pathways are distinct (7, 18). This may suggest that STING was initially evolved to sense cyclic dinucleotides from bacteria and was later co-opted for DNA sensing of viruses and damaged self (19).

Structural analysis of STING has indicated that it exists as a highly structured dimer containing an extensive hydrophobic dimer interface (18). CDG binding induces an untwisting 180^o^ rotation of the ligand-binding domain relative to the transmembrane domain and the formation of an ordered β-sheet “lid” that covers the ligand-binding domain. These changes facilitate side-by-side oligomerization of STING dimers (9, 10, 16, 20). The STING polymer is covalently stabilized by disulfide bond formation between neighboring STING dimers through linkage at cysteine residues 148 (10). Consistently, a hyperactive form of STING known as SAVI (STING-associated vasculopathy with onset in infancy) carries mutations around cysteine 148 that enhance STING polymer formation and stabilization (10). Oligomerization is also fostered by palmitoylation of cysteine residues C88 and C91. Inhibition of STING palmitoylation at cysteines 88/91 inhibited STING polymerization and blocked STING signaling (21, 22). Notably, canonical cyclic dinucleotides and some small-molecule STING agonists promote STING oligomerization without the rearrangement of the lid region.

The carboxyl-terminal tail of STING is about 40 amino acids, resides in the cytoplasm, and interacts directly with both IRF3 and TBK1 for activation of downstream signaling (23). It has been proposed that the STING polymer scaffolds the interaction between IRF3 and TBK1. Furthermore, since IRF3 dimerization is important for STING signaling it has also been proposed that the STING polymer scaffolds IRF3 dimerization (23). IRF3 activation leads to type I interferon responses (8).

The localization of STING is important for signaling. Inactive STING dimers localize to the ER and the Stromal Interaction Molecule 1 (STIM1) is important for retaining STING in the ER (24). Binding of a ligand to STING promotes its translocation to the Golgi apparatus where palmitoylation occurs (21). Palmitoylation has been implicated in clustering of proteins into specific membrane domains enriched in cholesterol and sphingomyelin, known as lipid rafts (25–27). The palmitoylation of STING seems to be important for its oligomerization and the recruitment of downstream signaling factors (21, 22). STING can exit the Golgi and has been suggested to move to endosomes, to compartments positive for the autophagy adaptor protein SQSTM1/p62, and eventually to lysosomes for degradation (28). Autophagy-mediated degradation of STING could be a mechanism to negatively regulate its activity. The post-Golgi trafficking of STING remains uncharacterized.

In 2014, we published that during HSV-1 infection STING is released from infected cells in exosomes (5). We later found that EVs released from HSV-1-infected cells activated innate immune responses in uninfected recipient cells in a STING-dependent manner and suppressed HSV-1 infection (29). We have built upon these studies and have determined the STING exocytosis pathway during HSV-1 infection. We have found that HSV-1 infection stimulates the production of EVs through the CD63 tetraspanin exocytosis pathway. STING co-localized with CD63 in globular structures in the cytoplasm and co-fractionated with CD63 + EVs. STING exocytosis did not occur after infecting CD63 knockdown cells. These data indicate that STING is packaged in CD63 + EVs. The STING exocytosis pathway also involved trafficking through the Golgi since golgicide A and brefeldin A treatment blocked STING exocytosis after HSV-1 infection. STING ligands such as 2′3′-cGAMP or the genome of a replication-defective HSV-1 virus were not sufficient to cause STING exocytosis, rather replication/late gene expression during HSV-1 infection, and perhaps capsid assembly, were required. Also, STING downstream signaling was not required for its exocytosis. STING exocytosis was dependent on palmitoylation at cysteines 88/91 and on STIM1. Finally, we found that other herpes viruses such as VZV and HCMV promoted STING exocytosis, but not the HSV-2 strain G. Therefore, EVs from HSV-1- and HSV-2-infected cells differentially impacted these viruses, with HSV-1 EVs restricting both HSV-1 and HSV-2 infection, unlike EVs produced from HSV-2(G)-infected cells that displayed no effect on either virus infection.

Overall, during infection by herpes viruses, STING can exit the Golgi and enter in late endosomes carrying the CD63 tetraspanin. These endosomes mature into vesicles that will deliver STING to the extracellular space, where it alters the microenvironment of infection.

## RESULTS

### Depletion of CD63 inhibited STING exocytosis during HSV-1 infection

**Fig 1:**
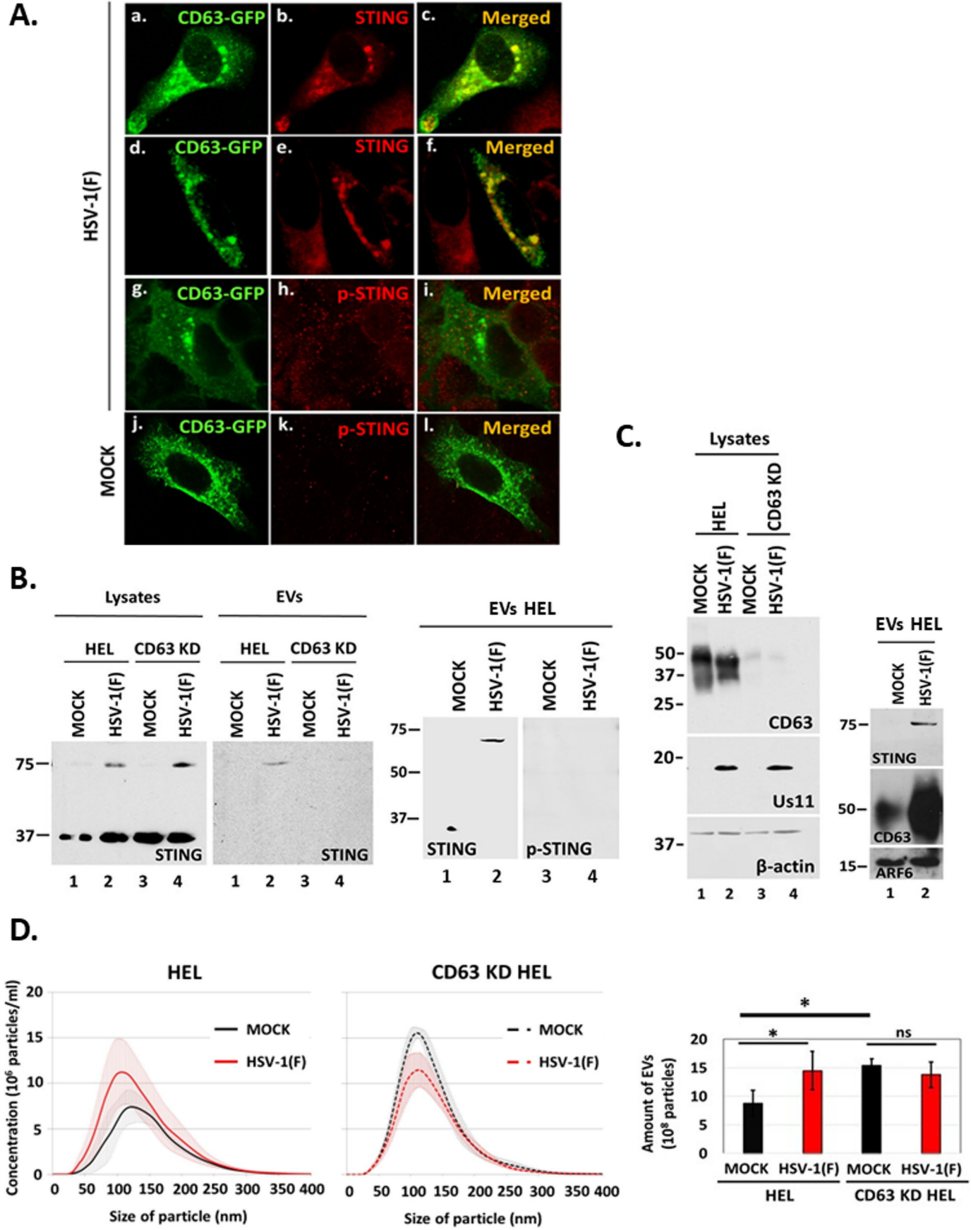
STING is packaged in CD63 + EVs during HSV-1 infection. (**A**) The Flag-STING-expressing HEp-2 cell line was transfected with a CD63-GFP-expressing plasmid (panels a–c). We also used replicate cultures of a CD63-GFP-expressing HEp-2 cell line (d–l) . At 24 h post-transfection, the cells were infected with HSV-1(F) (10 PFU/cell). The cells were fixed using 4% paraformaldehyde at 10 h post-infection and stained with an anti-Flag antibody (panels a–c), with an anti-STING antibody (panels d–f), or with a phospho-STING (Ser-366) antibody (panels g–l). Images were obtained using a Leica confocal microscope. (**B**) HEL cells and the CD63 KD derivatives were either uninfected or infected with HSV-1(F) (0.5 PFU/cell), and cell lysates and culture supernatants were harvested at 48 h post-infection. EVs were isolated from the supernatants after pelleting at 100,000 × *g*. Samples were analyzed by western blot for STING. EVs from HEL cells were also analyzed for phospho-STING (Ser-366). (**C**) HEL cells and the CD63 KD derivatives were infected with HSV-1(F) (1 PFU/cell). The cells were harvested at 48 h post-infection, and equal amounts of proteins from cell lysates were analyzed for CD63 expression and viral gene expression (Us11). B-actin served as a loading control. Parallel cultures of HEL cells were infected with HSV-1(F) (0.5 PFU/cell) and EVs that were pelleted from culture supernatant as above were analyzed for CD63, STING, and ARF6, a marker of microvesicles. (**D**) HEL cells and the CD63 KD derivatives were either uninfected or infected with HSV-1(F) (0.5 PFU/cell). Cell supernatants were harvested at 48 h post-infection, and EVs were isolated through an iodixanol-sucrose gradient and analyzed by nanoparticle tracking analysis (NTA) as described in Materials and Methods. Quantification of EVs from at least three independent experiments is depicted. *P  ≤  0.05; **P  ≤  0.01; ***P  ≤  0.001.

In our earlier studies, we demonstrated that HSV-1 infection stimulates the production of EVs through the CD63 exocytosis pathway, as well as STING exocytosis (30, 31). We also found that STING co-fractionated with CD63 + EVs in a 6%–18% iodixanol/sucrose gradient (31). To determine if STING and CD63 co-localize, HEp-2 cells constitutively expressing Flag-tagged STING were transfected with a plasmid expressing CD63 fused to GFP. At 24 h post-transfection, the cells were infected with HSV-1 and co-localization of STING with CD63 was determined by confocal microscopy. We observed that STING and CD63 co-localized in globular structures in the cytoplasm (Fig. 1A, panels a through c). Similar colocalization of endogenous STING with CD63-GFP was noticed in HEp-2-infected cells (Fig. 1A, panels d through f). Phosphorylation of STING on serine-366 is considered part of the STING activation process. We noticed no colocalization of phospho-STING (Ser-366) with CD63-GFP in infected or uninfected cells (Fig. 1A, panels g through l). To determine if STING exocytosis requires CD63, we infected HEL cells and CD63 knockdown derivatives with HSV-1(F) (0.5 PFU/cell). Culture supernatants were collected at 48 h post-infection, total EVs were isolated as we have described before, and the presence of STING in EVs was assessed by western blot (29, 31). STING was detected in EVs released from infected but not from uninfected HEL cells. STING was not detected in EVs released from infected CD63 knockdown cells although higher amounts of STING dimers were present intracellularly (Fig. 1B). Consistent with the immunofluorescence data, we did not detect phospho-STING (Ser-366) in EVs (Fig. 1B). This is likely because the virus efficiently counteracts antiviral responses in HEL cells, where it productively replicates. Intracellular levels of CD63 are usually reduced during HSV-1(F) infection, as the protein is exocytosed in EVs in large quantities (Fig. 1C) (29–31). As we have previously published, increased exocytosis through the CD63 pathway during HSV-1 infection was specific as indicated by the levels of ARF6, a marker of microvesicles, that were comparable between infected and uninfected cells (Fig. 1C) and by the levels of ESCRT components that we published before (29, 31, 32). Also, HSV-1 infection did not induce increased production of EVs in CD63 KD cells compared with uninfected CD63 KD cells (Fig. 1D) even though CD63 KD cells produced higher virus yields compared with parental cells (29). Notably, uninfected CD63 KD cells naturally produced more EVs compared with their parental cells, as they most likely activate alternative exocytosis pathways to compensate for the absence of exocytosis through the CD63 pathway (Fig. 1D). These alternative exocytosis pathways do not appear to support STING exocytosis (Fig. 1B). A quantification of EVs from infected and uninfected CD63 KD cells vs parental cells from at least three independent experiments is depicted (Fig. 1D). The efficiency of CD63 depletion is depicted in Fig. 1C. We conclude that STING exocytosis during HSV-1 infection requires CD63.

### STING exocytosis requires trafficking from the ER through the ERGIC to the TGN

**Fig 2:**
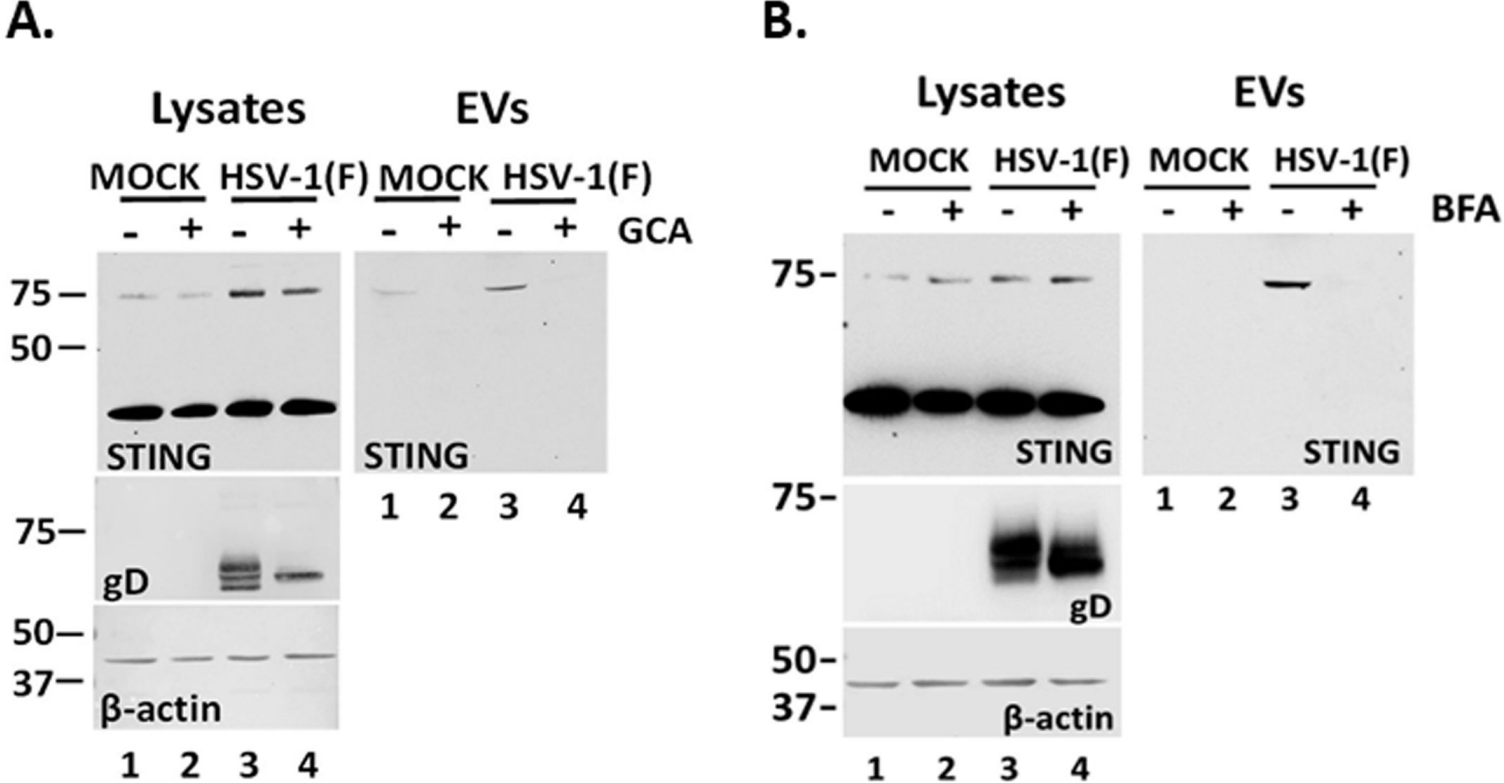
STING packaging in EVs requires Golgi integrity. HEL cells were either uninfected or infected with HSV-1(F) (0.5 PFU/cell) and either untreated or treated with (**A**) 10 µM golgicide A or (**B**) 2.5 µg/mL brefeldin A. Cell lysates and culture supernatants were harvested at 48 h post-infection. EVs were isolated from supernatants after pelleting at 100,000 × *g*. Samples were analyzed by western blot for STING, and β-actin served as a loading control. Probing for gD served as control for compound activity.

STING is a transmembrane protein in the ER that translocates to the TGN and perinuclear compartments following ligand binding (4). To determine whether STING translocation through the Golgi is important for STING exocytosis, we performed two sets of experiments. First, we infected HEL cells with HSV-1(F) (0.5 PFU/cell) and treated them with golgicide A (GCA) (10 µM). GCA is a highly specific and reversible inhibitor of the *cis*-Golgi ARF1 GEF GBF1 that is essential for the assembly of COP-I vesicles (33). Culture supernatants were collected at 48 h post-infection, and total EVs were analyzed for the presence of STING. Treated and untreated uninfected cells served as a control. As shown in Fig. 2A, HSV-1 infection triggered the formation of STING dimers in the presence or absence of GCA (lanes 3, 4 compared to lanes 1, 2). However, GCA blocked STING exocytosis from infected cells. We demonstrated that GCA is active since it prevented glycoprotein D (gD) glycosylation during HSV-1 infection by blocking its trafficking through the Golgi. Second, a similar analysis was performed using brefeldin A (BFA) (2.5 µg/mL). BFA is an inhibitor of different ADP ribosylation factors (Arfs) and causes Golgi disassembly (34). Like GCA, BFA disrupted STING exocytosis (Fig. 2B, EVs, compare lane 4 to lane 3) and also interfered with gD glycosylation (Fig. 2B, lysates, compare lane 4 to lane 3). We conclude that trafficking through the Golgi is essential for STING to enter CD63 + multivesicular bodies (MVBs).

### The STING ligand 2′3′-cGAMP and TBK1 STING signaling do not trigger STING exocytosis

**Fig 3:**
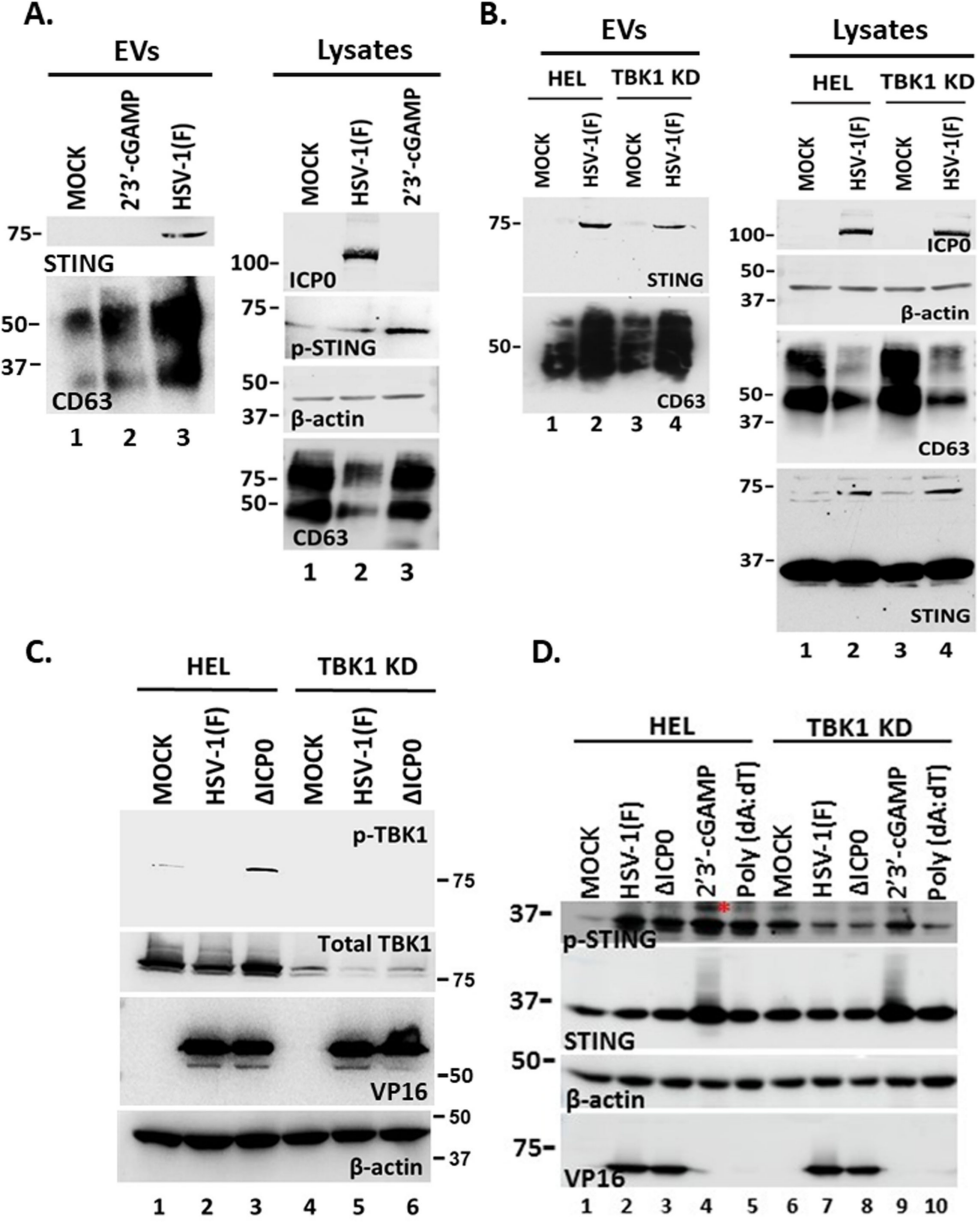
STING exocytosis is triggered by HSV-1(F) infection but not by a STING ligand and downstream effector binding. (**A**) HEL cells were uninfected, transfected with 2′3′-cGAMP at a final concentration of 3 µM, or infected with HSV-1(F) at 0.5 PFU/cell. EVs were isolated from the supernatant after pelleting at 100,000 × *g* and analyzed by western blot for STING or CD63. Also, equal amounts of proteins from total cell lysates were analyzed for CD63 and p-STING. ICP0 served as a control for infection and β-actin served as a loading control. (**B**) HEL cells and their TBK1 knockdown derivatives were either uninfected or infected with HSV-1(F) (0.5 PFU/cell). Culture supernatants were harvested at 48 h post-infection, and EVs were isolated after centrifugation at 100,000 × *g*. Samples were analyzed by western blot for STING and CD63. In addition, equal amounts of proteins from total cell lysates were analyzed for STING and CD63. ICP0 served as a control of infection and β-actin as a loading control. (**C**) HEL cells and the TBK1 KD derivatives were either uninfected or infected with HSV-1(F) or ΔICP0 virus (0.5 PFU/cell). Cell lysates were harvested at 48 h post-infection and analyzed for p-TBK1 (Ser-172) and total TBK1. Probing for β-actin served as a loading control and for VP16 as a control for the infection. (**D**) HEL cells and the TBK1 KD derivatives were uninfected, infected with HSV-1(F), ΔICP0 virus (0.5 PFU/cell), or transfected with 2′3′-cGAMP (6 µM). The cells were harvested at 48 h post-treatment or post-infection, and equal amounts of proteins from total cell lysates were analyzed for p-STING (Ser-366) and STING. VP16 served as a control for the infection and β-actin as a loading control. Red asterisk marks p-STING (Ser-366).

To determine if activation of STING through ligand binding triggers STING exocytosis, we treated HEL cells with the STING ligand 2′3′-cGAMP (3 µM) for 48 h. EVs were isolated from the supernatant of 2′3′-cGAMP-treated, untreated, and HSV-1(F)-infected HEL cells, and exocytosis of STING and CD63 was determined by western blot (Fig. 3). The ligand 2′3′-cGAMP alone did not trigger STING exocytosis compared with infected cells even though it caused STING activation, as indicated by the increased levels of STING phosphorylation at serine-366 (Ser-366) (Fig. 3A). Consistently, CD63 exocytosis was not stimulated in 2′3′-cGAMP-treated cells compared with HSV-1(F)-infected cells (Fig. 3A). As discussed above, in cell lysates, we observed a decrease in the amounts of CD63 during HSV-1 infection due to increased exocytosis, but not after treatment with 2′3′-cGAMP (Fig. 3A) (29–31). Thus, a STING ligand alone could not trigger STING or CD63 exocytosis. We also asked if downstream STING signaling was required for STING exocytosis. TBK1 is a major effector molecule that conveys signals from activated STING to the nucleus for activation of type I interferon responses. We found that exocytosis of STING dimers was comparable between infected HEL cells and the TBK1 KD derivatives, and the levels of intracellular STING dimers were also comparable (Fig. 3B). Also, CD63 exocytosis was comparable between infected TBK1 KD HEL cells and parental cells (Fig. 3B). Consistently, we noticed a comparable decrease of intracellular CD63 between infected HEL cells and TBK1 KD derivatives. The efficiency of TBK1 depletion is depicted in Fig. 3C. As a control, we verified that the TBK1 KD HEL cells were deficient in activation of downstream signaling as we did not detect p-TBK1 (Ser-172) after infection with an ICP0-null virus (ΔICP0) that has reduced ability to counteract type I IFN responses (Fig. 3C). Also, we verified that in TBK1 KD HEL cells phosphorylation of STING on Ser-366 could not be performed following treatment with the STING ligand 2′3′-cGAMP (Fig. 3D). We conclude that downstream STING signaling is not required for STING exocytosis in EVs.

### STING exocytosis occurs after infection with other herpesviruses and depends on virus replication/late gene expression

**Fig 4:**
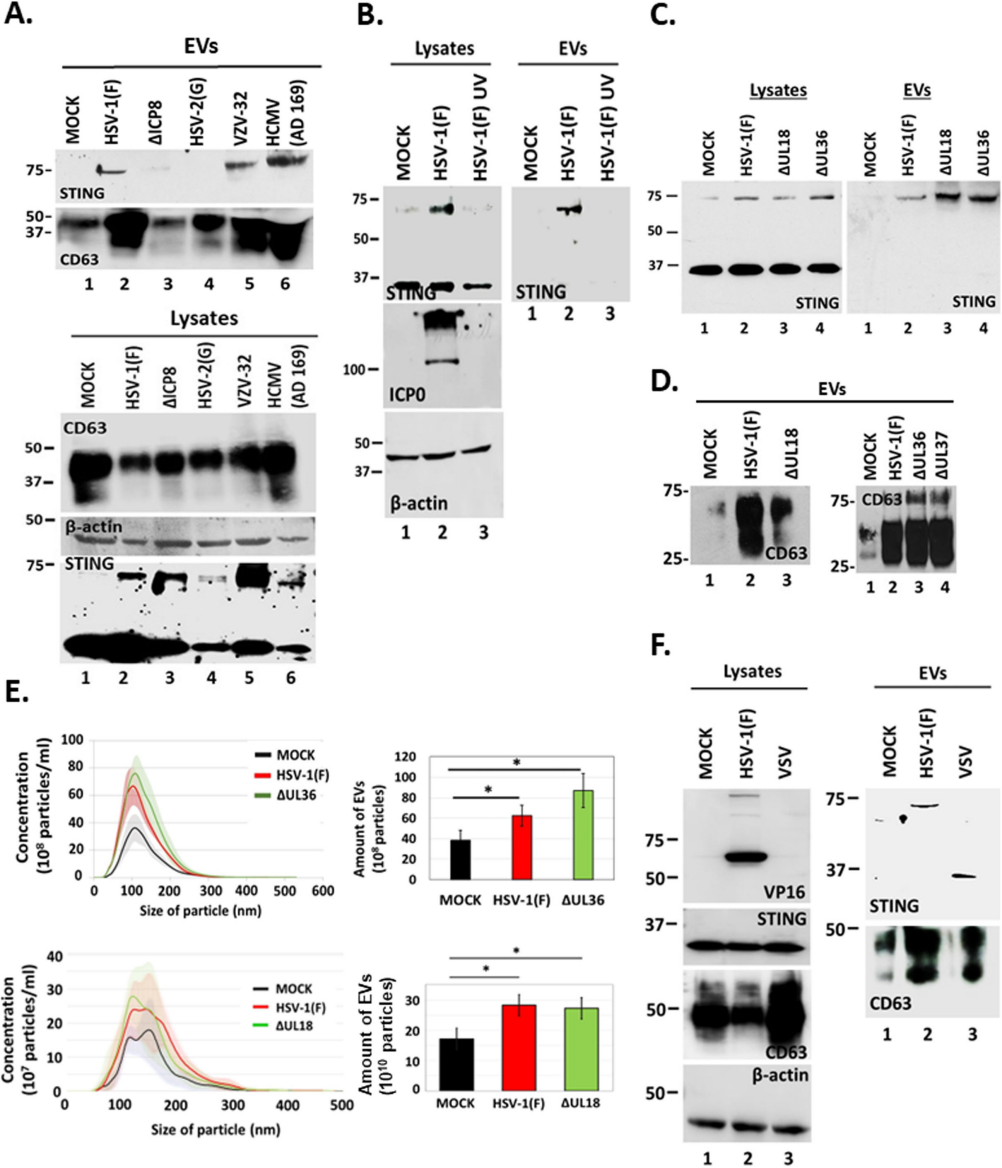
HSV-1 replication/late gene expression is required for CD63 and STING exocytosis. (**A**) HEL cells were either uninfected or infected with HSV-1(F), ΔICP8, HSV-2(G), VZV, or HCMV (0.5 PFU/cell). EVs were isolated from culture supernatants at 48 h post-infection or when CPEs were comparable to HSV-1(F) at 48 h post-infection by pelleting at 100,000 × *g*. EVs and total cell lysates were analyzed by western blot for STING and CD63. As a loading control, we used β-actin. (**B**) HEL cells were uninfected, infected with HSV-1(F) (0.5 PFU/cell), or with UV-inactivated virus (0.5 PFU/cell equivalent). Culture supernatants were harvested at 48 h post-infection, and EVs were analyzed for STING. Equal amounts of cell lysates were also analyzed for STING. ICP0 served as a control for infection and β-actin as a loading control. (**C and D**) HEL cells were either uninfected or infected with HSV-1(F), ΔUL18, ΔUL36, or ΔUL37 (0.5 PFU/cell). Cell lysates and culture supernatants were harvested at 48 h post-infection, and equal amounts of proteins from total cell lysates were analyzed for STING expression. EVs were isolated from the supernatant after centrifugation at 100,000 × *g*. Total EVs were analyzed by western blot for STING and CD63. (**E**) HEL cells were either uninfected or infected with HSV-1(F), ΔUL18, or ΔUL36 (0.5 PFU/cell). Cell supernatants were harvested at 48 h post-infection, EVs were isolated through an iodixanol-sucrose gradient and analyzed by NTA as described in Materials and Methods. Quantification of EVs from three independent experiments has been included. *P  ≤  0.05; **P  ≤  0.01; ***P  ≤  0.001. (**F**) HEL cells were uninfected or infected with HSV-1(F) (0.1 PFU/cell) or with vesicular stomatitis virus (VSV) (0.01 PFU/cell). Culture supernatants were collected when cytopathic effects were advanced (at 48 and 24 h, respectively), EVs were isolated as in panels C and D and analyzed for STING and CD63. Equal amounts of proteins from total cell lysates were also analyzed for CD63 and STING. VP16 was used as a control for HSV-1(F) infection and β-actin as a loading control.

To determine whether STING exocytosis occurs with other human herpesviruses, we infected HEL cells with HSV-1(F), HSV-2(G), VZV, or HCMV. Supernatant from HSV-1(F)- and HSV-2(G)-infected cultures was collected at 48 h post-infection. For VZV and HCMV, EVs were collected when we observed cytopathic effects, to the same extent, as HSV-1 since these viruses displayed a prolonged life cycle in HEL cells compared with HSV-1 and HSV-2. In this case, the culture medium was replaced 48 h before harvesting the EVs. Detection of STING in the 100,000 × *g* pellet of EVs was performed by western blot. STING exocytosis was observed after infection with HSV-1(F), VZV, and HCMV but surprisingly not after infection with HSV-2(G) (Fig. 4A). CD63 exocytosis was triggered after infection with VZV and HCMV similar to HSV-1(F), but, to a lesser extent, after infection with HSV-2(G), which is consistent with our finding that STING is packaged in CD63 + EVs (Fig. 4A). STING dimers were detected in the lysates of the infected cells, but the levels of both the STING dimers and monomers varied suggesting that the different viruses had different effect on STING accumulation/stability (Fig. 4A). The levels of intracellular CD63 also varied and reversibly correlated with the levels of extracellular CD63 (Fig. 4A). Previously, we have determined that a replication-deficient virus ΔICP8 could not induce CD63exocytosis (30). Consistently, we observed that a ΔICP8 virus did not trigger STING exocytosis (Fig. 4A). Furthermore, we found that STING exocytosis did not occur following exposure of cells to UV-inactivated virus (Fig. 4B).

To further narrow down the stage of HSV-1 infection that triggers STING exocytosis, we infected HEL cells with either a capsid assembly-deficient virus ΔUL18 or an envelopment-deficient virus ΔUL36. STING exocytosis occurred after infection with either ΔUL18 or ΔUL36 viruses (Fig. 4C). CD63 exocytosis was reduced in ΔUL18 virus-infected cells compared with WT virus-infected cells but remained at levels similar to WT-virus in ΔUL36 and ΔUL37 virus-infected cells (Fig. 4D). EV production between ΔUL36 and WT-virus, as well as ΔUL18 and WT-virus, was indistinguishable and was higher than for uninfected cells (Fig. 4E). A quantification of EVs from at least three independent experiments is depicted in Fig. 4E. Finally, we determined whether STING exocytosis was specific for herpesviruses or other viruses could also trigger STING exocytosis. For this, HEL cells were exposed to vesicular stomatitis virus (VSV), an RNA virus, and EVs were collected when extensive cytopathic effects were apparent and compared with EVs from HSV-1(F)-infected cells for STING and CD63. While HSV-1(F) infection caused exocytosis of STING dimers, in VSV-infected cells, we observed exocytosis of STING monomers but not of STING dimers (Fig. 4F). Exocytosis of CD63 was comparable between cells infected with these viruses (Fig. 4F). VSV caused an increase in the levels of intracellular CD63 compared with uninfected and HSV-1(F)-infected cells and increased CD63 exocytosis compared with uninfected cells (Fig. 4F). However, it is currently unknown if VSV promotes STING exocytosis via the same pathway as HSV-1(F). We conclude that STING exocytosis follows CD63 exocytosis, and in the case of HSV-1, it depends on virus replication/late gene expression.

### STING dimers were not exocytosed after infection with HSV-2(G)

**Fig 5:**
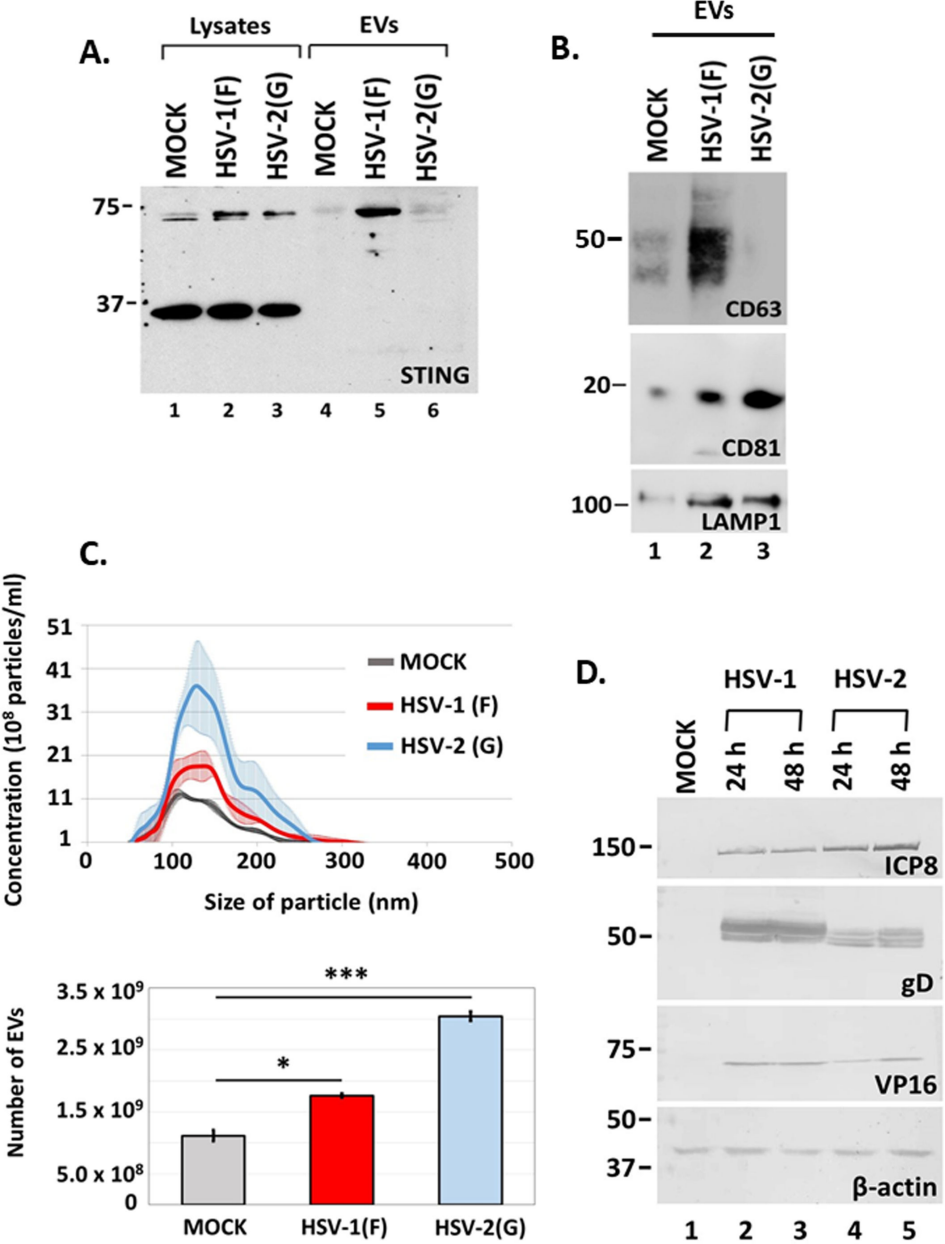
Differences in STING and CD63 exocytosis between HSV-1(F) and HSV-2(G)-infected cells. (**A and B**) HEL cells were either uninfected or infected with HSV-1(F) or HSV-2(G) at 0.5 PFU/cell. EVs were isolated from cell supernatants at 48 h post-infection by pelleting at 100,000 × *g*. Total EVs were analyzed by western blot for STING, CD63, CD81, and LAMP1. Detection of STING monomers and dimers in equal amounts of cell lysates served as a control. (**C**) HEL cells (1 × 10^8^) were either uninfected or infected with HSV-1(F) or HSV-2(G) at 0.5 PFU/cell. Cell supernatants were harvested at 48 h post-infection and EVs were isolated through an iodixanol-sucrose gradient and analyzed by NTA as described in Materials and Methods. All values were derived after analyzing samples from three independent experiments. *P  ≤  0.05; **P  ≤  0.01; ***P  ≤  0.001. (D) HEL cells were uninfected or infected with either HSV-1(F) or HSV-2(G) at 0.5 PFU/cell. Cell lysates were harvested at 24 and 48 h post-infection. Equal amounts of proteins were analyzed by western blot for the viral proteins ICP8, gD, and VP16. B-actin was used as a loading control.

While most herpesviruses induced exocytosis of STING dimers after infecting HEL cells, HSV-2(G) did not although STING dimers were present in lysates from HSV-2(G)-infected HEL cells in levels comparable to HSV-1(F)-infected cells at 48 h post-infection (Fig. 5A). We also determined whether HSV-2(G) could trigger CD63 exocytosis-like HSV-1(F). Culture supernatants were collected from uninfected and HSV-2(G)- or HSV-1(F)-infected HEL cells (0.5 PFU/cell) at 48 h post-infection, and the amounts of CD63 in isolated EVs were assessed by western blot. While HSV-1 induced CD63 exocytosis, HSV-2 did not (Fig. 5B). HSV-2(G) did not promote exocytosis through the ESCRT pathway either, rather it induced exocytosis of CD81, a tetraspanin that resides on the plasma membrane, and exocytosis of LAMP1, a late endosomal/lysosomal marker, to levels comparable to HSV-1(F) (Fig. 5B). HSV-2(G) induced production of EVs to levels comparable to HSV-1(F) as determined by nanoparticle tracking analysis (Fig. 5C), but these EVs were largely CD63 negative. These differences were not due to differences in the kinetics of viral gene expression between the two viruses (Fig. 5D). Overall, HSV-2(G) did not induce CD63 and consequently STING exocytosis to the same extent as HSV-1(F).

### STING palmitoylation is critical for its exocytosis during HSV-1 infection

**Fig 6:**
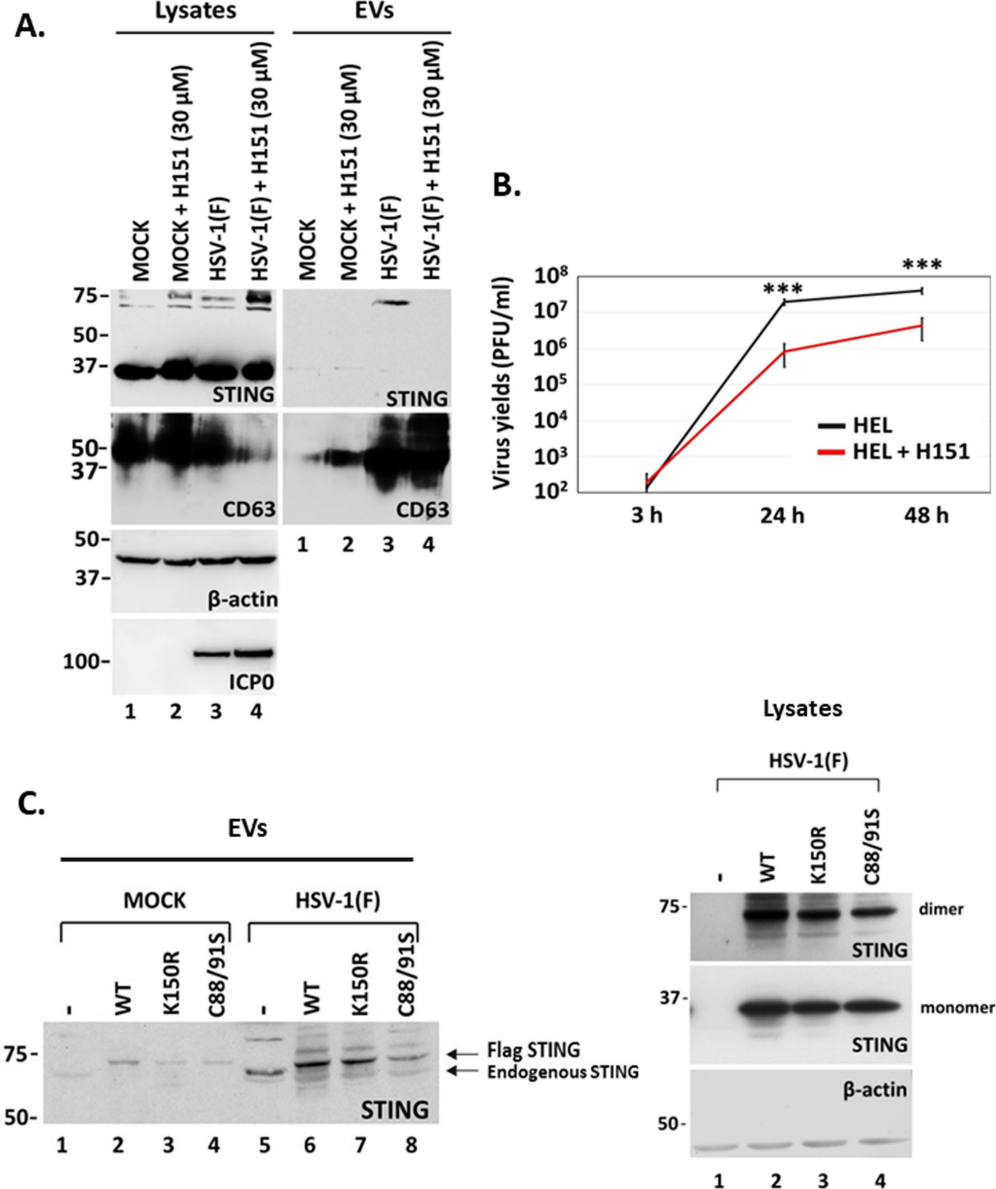
STING palmitoylation mediates STING exocytosis in EVs. (**A**) HEL cells were either uninfected or infected with HSV-1(F) (0.5 PFU/cell) and untreated or treated with H-151 (30 µM) that was added to the culture with the virus. Cell lysates and supernatants were harvested at 48 h post-infection, and EVs were isolated from the supernatants after pelleting at 100,000 × *g*. Total EVs were analyzed by western blot for STING and CD63. Also, proteins from equal amounts of cell lysates were analyzed for STING, CD63. ICP0 served as a control for infection and β-actin as a loading control. (**B**) HEL cells were infected with HSV-1(F) (0.5 PFU/cell), and untreated or treated with H-151 (30 µM) that was added to the cultures with the virus. Cells were collected at 3, 24, and 48 h post-infection, and progeny virus production was determined by plaque assays. All values were derived after analyzing samples from three independent experiments. *P  ≤  0.05; **P  ≤  0.01; ***P  ≤  0.001. (**C**) HEp-2 cells and derivatives expressing either unmodified Flag-tagged STING or Flag-tagged STING mutants were either uninfected or infected with HSV-1(F) (0.5 PFU/cell). Culture supernatants were harvested at 48 h post-infection, and EVs were isolated after pelleting at 100,000 × *g*. Total EVs were analyzed by western blot for STING. Equal amounts of cell lysates from infected cells were analyzed for STING monomers and dimers and for β-actin.

A common feature of STING and CD63 is the palmitoylation that facilitates their oligomerization. CD63 palmitoylation leads to the formation of tetraspanin-enriched microdomains, which laterally organize membranes via specific associations between tetraspanins and adhesion receptors (25). We hypothesized that STING palmitoylation is important for its exocytosis, as it could facilitate the association of STING with CD63 oligomers during HSV-1 infection. For this, we treated HSV-1-infected cells with H151, a highly potent and selective small-molecule antagonist of STING. H151 binds to STING through a covalent bond that is formed with Cys91 of STING, followed by an intramolecular rearrangement (22). HEL cells either uninfected or infected with HSV-1(F) (0.5 PFU/cell) were treated or not with H151 (30 µM). Culture supernatants were collected at 48 h post-infection, EVs were pelleted at 100,000 × *g,* and STING in EVs was detected by western blot. Analysis of STING, CD63, ICP0, and β-actin levels in equal amounts of cell lysates served as a control. STING dimers were present in lysates from HSV-1(F)-infected cells, and their amounts were increased in the presence of H151 (Fig. 6A, lysates, compare lane 4 to lane 3 and lane 2 to lane 1). However, STING exocytosis was observed only in HSV-1(F)-infected cells, but not in the presence of H151 (Fig. 6A, EVs, compare the panel of EVs, lane 4 to lane 3). Lack of STING exocytosis in infected, H151-treated cells was not due to the inhibition of CD63 exocytosis. As observed in Fig. 6A, in the presence of H-151, there was a greater decrease of intracellular CD63 in infected cells (lysates, lane 4) compared with infected, untreated cells (lysates, lane 3) followed by greater CD63 exocytosis (EVs, compare lane 4 to lane 3). H151 caused an approximately 10-fold decrease in HSV-1 progeny virus (Fig. 6B), but this did not interfere with virus-induced CD63 exocytosis that could affect STING exocytosis. In a complementary approach, we established three HEp-2 cell lines expressing unmodified Flag-tagged STING, or Flag-tagged STING mutants with substitutions at a ubiquitination site (K150R), or in two palmitoylation sites (C88/91S) (21, 35). HEp-2 cells and the derivatives expressing the different STING forms remained uninfected or infected with HSV-1(F) (0.5 PFU/cell). EVs were isolated from culture supernatant at 48 h post-infection and analyzed for STING exocytosis by western blot. Dimerized STING was enriched in EVs derived from infected cells expressing either endogenous STING or Flag-tagged STING (Fig. 6C). STING exocytosis was not disrupted by the K150R substitution (Fig. 6C, compare lane 7 to lane 6) but was decreased by the C88/91S substitutions that inhibited STING palmitoylation (Fig. 6C, compare lane 8 to lane 6). STING dimers were abundant in lysates of infected cells expressing exogenously these Flag-tagged forms of STING (Fig. 6C, lysates). We conclude that STING palmitoylation is important for its exocytosis during HSV-1 infection.

### STIM1 has a contributing role in STING exocytosis during HSV-1 infection

**Fig 7:**
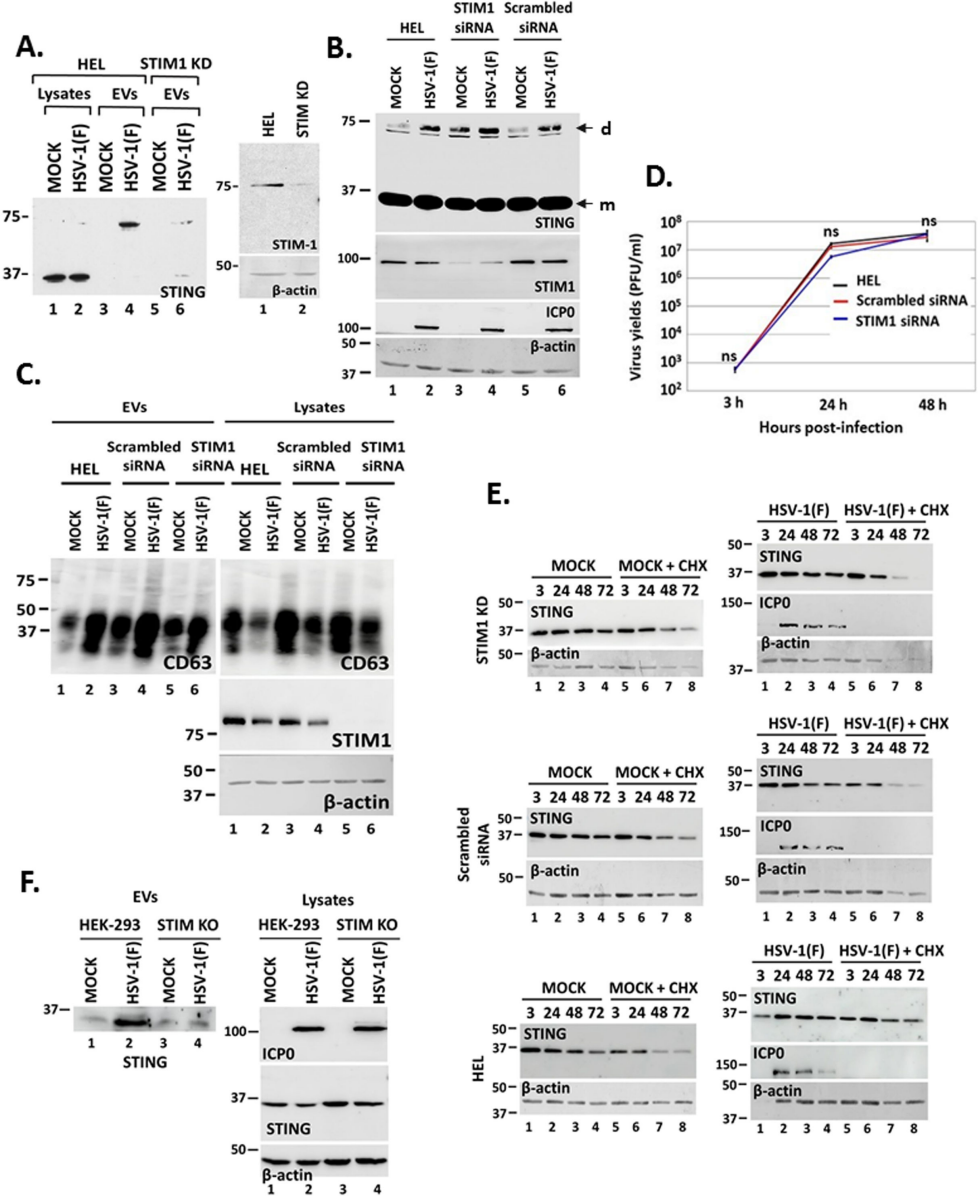
STING tethering to the ER by STIM1. (**A**) HEL cells and the STIM1 KD derivatives were either uninfected or infected with HSV-1(F) (0.5 PFU/cell). Cell lysates and culture supernatants were harvested at 48 h post-infection, and equal amounts of proteins from cell lysates were analyzed for STING expression. EVs were isolated from the supernatant after centrifugation at 100,000 × *g*. Total EVs were analyzed by western blot for STING. The efficiency of STIM1 depletion by a specific shRNA expressed from an integrated lentiviral vector following puromycin selection is depicted. (**B and C**) HEL cells either untreated, transfected with a scrambled siRNA, or with the STIM1 siRNA were infected with HSV-1(F) (0.5 PFU/cell) at 72 h post-transfection. Culture supernatant and cell lysates were harvested at 48 h post-infection and analyzed for CD63. Also, equal amounts of proteins from total cell lysates were analyzed for STING, STIM1, and ICP0. B-actin served as a loading control. Arrows indicate STING monomers (m) and STING dimers (d). (**D**) HEL cells and cells treated either with STIM1 siRNA or a control scrambled siRNA were infected with HSV-1 (0.01 PFU/cell). Samples were harvested at 3, 24, and 48 h post-infection, and quantification of progeny virus was done by plaque assays in Vero cells. This analysis was performed using three replicate samples from independent experiments. Differences were not statistically significant (ns). (**E**) HEL cells and cells treated either with STIM1 siRNA or a control scrambled siRNA were either uninfected or infected with HSV-1 (1 PFU/cell). Cycloheximide was added to the cultures at 3 h post-infection, and samples were harvested at 3, 24, 48, and 72 h post-infection. Equal amounts of proteins were analyzed for STING protein levels by western blot. ICP0 served as a control of infection and β-actin as a loading control. The efficiency of STIM1 depletion by the siRNA is depicted in panel C. (**F**) HEK-293 and STIM KO derivatives were uninfected or infected with HSV-1(F) (0.1 PFU/cell). Culture supernatants were collected at 36 h post-infection, and isolated EVs were analyzed for STING exocytosis. ICP0 served as a control for infection and β-actin as a loading control.

STIM1 localizes in the ER membranes and appears to tether STING to the ER to prevent constitutive STING activation (24). To determine if STIM1 is implicated in STING exocytosis, we developed a STIM1 KD cell line and assessed STING exocytosis at 48 h following HSV-1 infection (0.5 PFU/cell). STING exocytosis was observed after infecting the parental cells, but reduced STING exocytosis was observed after infecting STIM1 KD derivatives (Fig. 7A). The efficiency of STIM1 depletion in HEL cells is depicted in Fig. 7A. Depletion of STIM1 did not interfere with STING dimerization during HSV-1 infection; thus, higher amounts of STING dimers (d) were observed in uninfected, STIM1 depleted cells (Fig. 7B, compare lane 3 to lanes 1 and 5). We also determined if STIM1 depletion affects CD63 exocytosis. For this, we compared the levels of exosomal and intracellular CD63 between uninfected and infected cells that were either untreated, treated with a scrambled siRNA, or with a STIM1 siRNA. STIM1 depletion did not alter the ability of the virus to induce CD63 exocytosis, as indicated by the levels of exosomal CD63, which were increased during HSV-1 infection, and the levels of intracellular CD63, which were decreased during HSV-1 infection (Fig. 7C). We also determined that STIM1 depletion did not affect HSV-1(F) progeny virus production compared with untreated or control siRNA treated cells (Fig. 7D). As an additional control, we determined whether STING stability was affected in STIM1 KD cells. Replicate cultures of HEL, STIM1 KD, and control siRNA-treated cells were either uninfected or infected with HSV-1(F) (1 PFU/cell). Cycloheximide (100 µg/mL) was added to the cultures at 3 h post-infection, and samples were harvested at 3, 24, 48, and 72 h after infection. We found that STING stability was comparable between infected and uninfected HEL cells and the STIM1 KD or control siRNA-treated cells (Fig. 7E). Finally, we compared STING exocytosis after infecting HEK-293 STIM double KO cells with HSV-1 (F) (0.1 PFU/cell). We observed decreased STING exocytosis by infected STING KO versus parental cells (Fig. 7F). We conclude that STIM1 has a role in STING exocytosis during HSV-1 infection.

### HSV-1(F) EVs were restrictive but HSV-2(G) EVs were not

**Fig 8:**
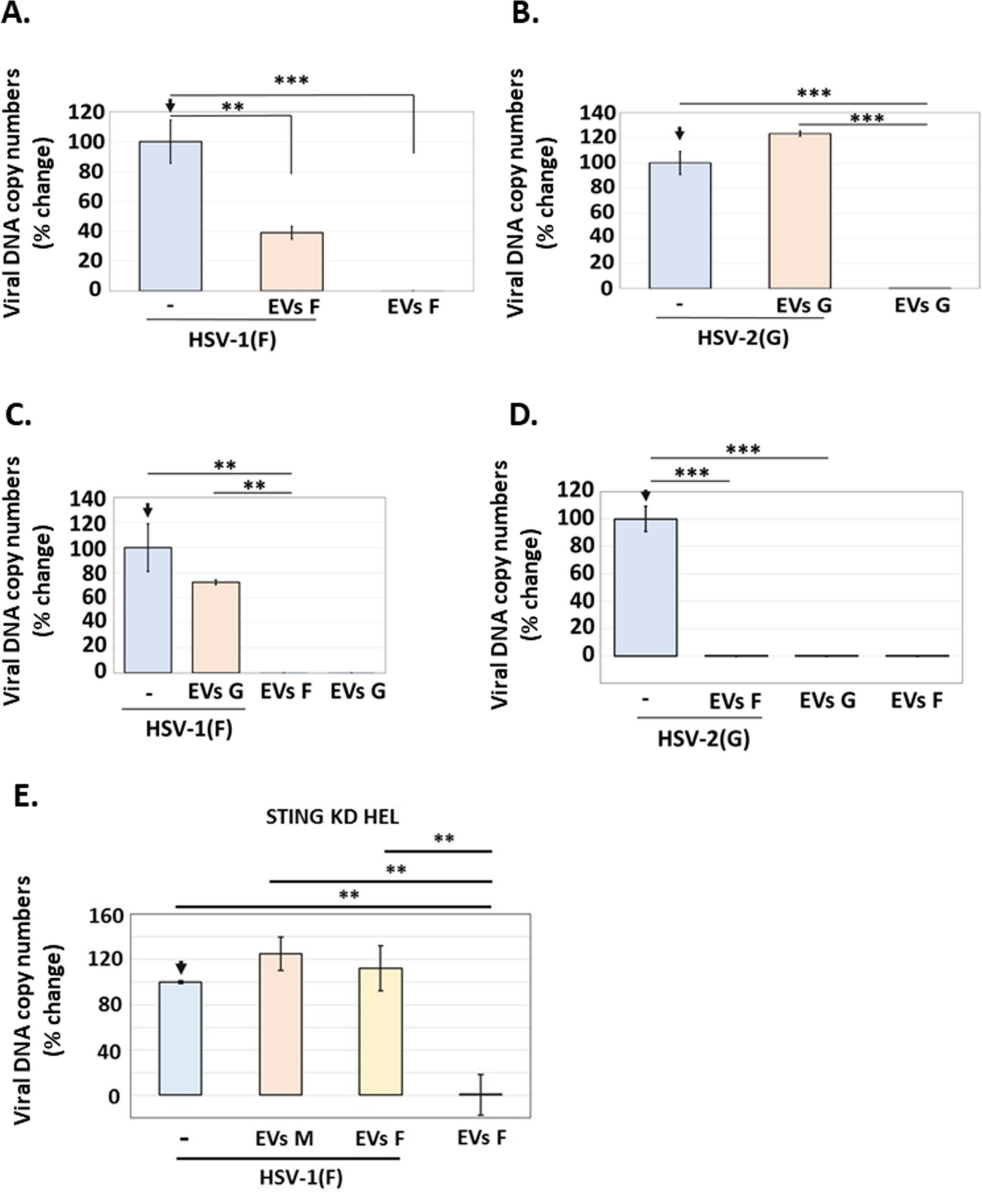
EVs from HSV-1(F)-infected cells inhibited infection by HSV-1(F) or HSV-2(G), while EVs from HSV-2(G)-infected cells had no effect on HSV-1(F) or HSV-2(G). (**A and B**) EVs from HSV-1(F)- (**A**) or HSV-2(G)- (**B**) infected HEL cells (0.1 PFU/cell) were harvested at 24 h post-infection. EVs were separated from virus using the discontinuous sucrose-iodixanol gradient previously developed by our laboratory (29–31). Then, fractions 4–8, previously identified by our lab to contain CD63 + EVs, were collected, and EVs numbers were quantified by NTA. An equal volume of EVs was then used to expose HEL cells, corresponding to approximately 2,000 EVs/cell from HSV-1(F) and 3,000 EVs/cell from HSV-2(G). At 2 h post-EV exposure, cells were either left uninfected or infected with HSV-1(F) (**A**) or HSV-2(G) (**B**) at 0.01 PFU/cell. The cells were harvested at 24 h post-infection, and quantification of the viral genome was done by qPCR analysis as detailed in the Materials and Methods. (**C and D**) EVs were harvested and quantified from HSV-1(F)- or HSV-2(G)-infected HEL cells as described in A and B. HEL cells were then pre-exposed to EVs from HSV-1(F) or HSV-2(G) for 2 h, and then, cells were either left uninfected or infected with HSV-1(F) (0.01 PFU/cell) (**C**) or HSV-2(G) (0.01 PFU/cell) (**D**) to assess the effect of HSV-2(G) EVs on HSV-1(F) infection (**C**) or the effects of HSV-1(F) EVs on HSV-2(G) infection (**D**). At 24 h post-infection, the cells were harvested, and quantification of the viral genome was done by qPCR analysis as detailed in the Materials and Methods. (**E**) EVs were harvested and quantified from HSV-1(F)-infected STING KD HEL cells as described in A and B. Equal volume of EVs was then used to expose HEL cells, corresponding to approximately 2,000 EVs/cell from HSV-1(F) and 1,000 EVs/cell from uninfected cells. At 2 h post-EV exposure, cells were either left uninfected or infected with HSV-1(F) at 0.01 PFU/cell. The cells were harvested at 24 h post-infection, and quantification of the viral genome was done by qPCR analysis as detailed in the Materials and Methods. *P  ≤  0.05; **P  ≤  0.01; ***P  ≤  0.001.

Previously, we determined that the dominant population of EVs released by HSV-1-infected cells is through the CD63 biogenesis pathway and that these EVs had a negative effect on HSV-1 infection (29–31). Moreover, we found that this negative effect of EVs was STING-dependent as EVs released by infected STING KD cells failed to restrict HSV-1 infection (29–31). Given the substantial differences between EVs released by HSV-1(F)- versus HSV-2(G)-infected cells, we sought to determine their ability to restrict these viruses. Consistent with our previous studies, we found that HSV-1(F) EVs had a negative effect on HSV-1(F) infection, as cells pre-treated with HSV-1(F) EVs followed by HSV-1(F) infection displayed an ~70% reduction in HSV-1(F) replication (Fig. 8A). In contrast, EVs released by HSV-2(G)-infected cells did not affect HSV-2(G) replication (Fig. 8B). A striking finding was that EVs released by HSV-1(F)-infected cells caused an ~1,000-fold decrease in HSV-2(G) replication, while equal amounts of HSV-2(G) EVs had no effect on HSV-1(F) replication (Fig. 8C and D). To determine if the restrictive effect of HSV-1(F) is due to STING and/or STING-dependent cargo, we isolated EVs from HSV-1(F)-infected and uninfected STING KD HEL cells and exposed them to HEL cells followed by HSV-1(F) infection. Unlike the EVs from infected, STING-expressing cells, the EVs from infected STING KD cells did not display a restrictive effect on HSV-1(F) infection (Fig. 8E).

**Fig 9:**
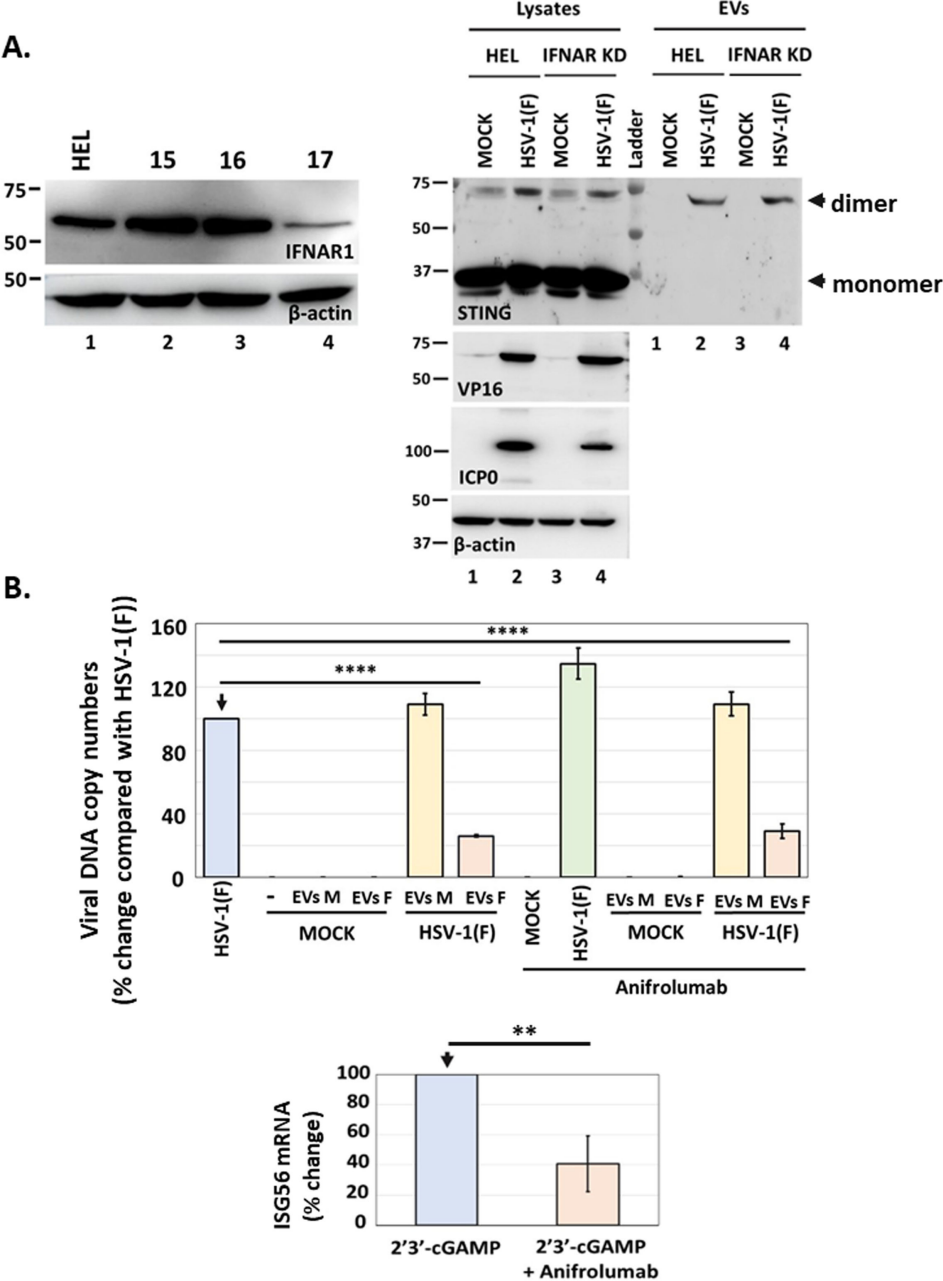
Effect of IFNAR1 on STING exocytosis and the antiviral function of EVs. (**A**) HEL cells and the IFNAR1 KD derivatives were either uninfected or infected with HSV-1(F) (0.5 PFU/cell). Cell lysates and culture supernatants were harvested at 48 h post-infection, and equal amounts of proteins from cell lysates were analyzed for STING expression. EVs were isolated from the supernatant after centrifugation at 100,000 × *g*. Total EVs were analyzed by western blot for STING. VP16 and ICP0 were used as controls for the infection and β-actin as a loading control. The efficiency of IFNAR1 depletion by different shRNAs expressed from an integrated lentiviral vector following puromycin selection is depicted. More efficient IFNAR1 depletion was achieved by shRNA #17. (**B**) EVs were produced and isolated from uninfected and HSV-1(F)-infected HEL cells as in Fig. 8A. Replicate cultures of HEL cells were treated or not with anifrolumab (1 µg/mL), an IFNAR1 neutralizing for 2 h. The cells were then treated or not with EVs from uninfected (3,000 EVs/cell) or EVs from HSV-1(F)-infected cells (4,300 EVs/cell) for 3 h. EVs were washed, and the cells were infected or not with HSV-1(F) (0.01 PFU/cell). The cells were harvested at 24 h post-infection and analyzed for viral genome copy numbers as in Fig. 8. As a control, we transfected HEL cells with 2′3′-cGAMP (6 µM) in the presence or absence of anifrolumab (1 µg/mL). Cells were harvested at 24 h post-treatment, and ISG56 mRNA was quantified by RT-qPCR.

To further delineate the mechanism of restriction of HSV-1 by the STING-dependent cargo, we sought to determine the role of IFNAR1 (Interferon Alpha and Beta Receptor Subunit 1). Two sets of experiments were performed: first, we depleted cells of IFNRA1 and assessed for STING exocytosis. We found that STING dimers were still formed in HSV-1(F)-infected, IFNAR1 KD cells, and these dimers were exocytosed during HSV-1(F) infection (Fig. 9A). Second, we blocked IFNAR1 with anti-IFNAR1, exposed cells to EVs derived from HSV-1(F)-infected or uninfected cells, and determined their ability to restrict HSV-1(F) infection. We found that these EVs could still restrict HSV-1(F) infection (Fig. 9B). As a control, we determined that anti-IFNAR1 could restrict antiviral responses due to 2′3′-cGAMP treatment. We conclude that CD63 + EVs from HSV-1(F)-infected cells carrying STING could restrict both HSV-1(F) and HSV-2(G), albeit to different extents, whereas EVs from HSV-2(G)-infected cells lacking CD63 and STING displayed no effect on HSV-1(F) or HSV-2(G).

## DISCUSSION

**Fig 10:**
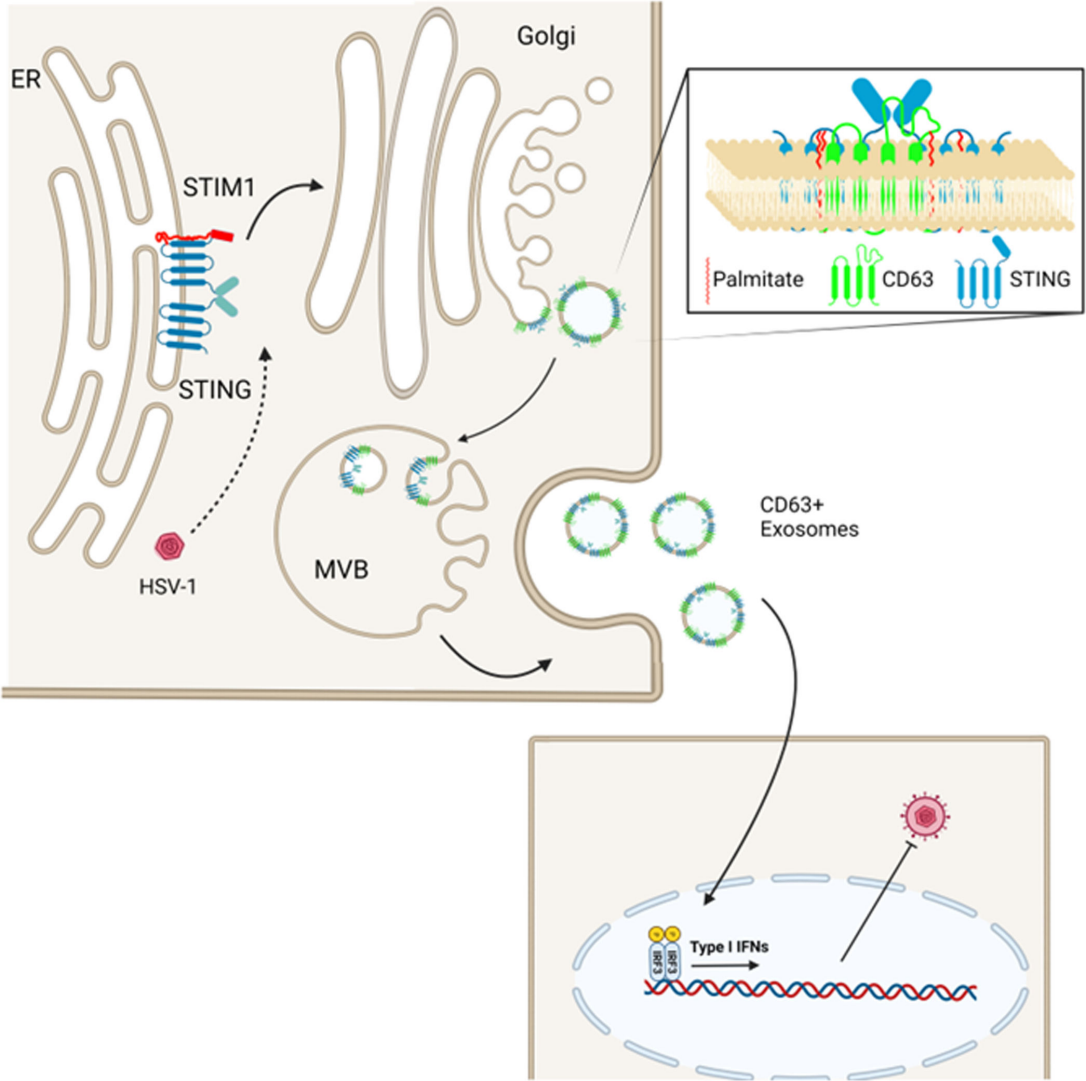
Model of STING exocytosis in EVs. STING is tethered to the ER through STIM1 and potentially other factors, but infection triggers conformational changes and translocation to the Golgi. From the Golgi, STING is transferred into CD63 + intraluminal vesicles (ILVs) of MVBs. CD63 + ILVs are released as CD63 + exosomes, and they have an antiviral effect in recipient cells, through stimulation of type I IFNs (29).

Previously, we demonstrated that the DNA sensor STING co-fractionated with CD63 + EVs, whose exocytosis was induced during HSV-1 infection (36). Furthermore, we have demonstrated that CD63 + EVs activated antiviral responses in uninfected recipient cells and restricted HSV-1 infection in a STING-dependent manner (29, 36). This restrictive effect of EVs and activation of type I IFN responses in recipient cells seems counterintuitive given that the virus can efficiently counteract type I IFN responses in the EV producer cells where it productively replicates. We have expanded upon these findings by tracing the STING exocytosis pathway. The salient features of our study can be summarized as follows (Fig. 10): STING is tethered to the ER by STIM1 and other factors, where it can be detected as a dimer during HSV-1 infection. Translocation of STING to CD63 + vesicles requires trafficking through the Golgi and palmitoylation of STING at residues C88/91. The ligand of STING 2′3′-cGAMP, foreign DNA such as from the replication-deficient HSV-1 virus ΔICP8 and from a UV-inactivated virus, was not sufficient to trigger STING exocytosis. Also, STING exocytosis was not dependent on signaling through its downstream effector TBK1. Increased STING dimerization was not sufficient for STING exocytosis as STING dimers were present in cells treated with golgicide A, brefeldin A or infected with HSV-2(G) but were not sorted in EVs. However, STING dimers were released during VZV and HCMV infections along with CD63 + EVs, as in HSV-1-infected cells, suggesting that STING exocytosis is common among herpesviruses despite a few exceptions. Finally, our data indicated that STING exocytosis in immortalized fibroblasts is a virus-induced process that requires virus replication/late gene expression and perhaps is a counter-reaction of the virus to host responses. When we analyzed RNA viruses such as VSV for STING exocytosis, we noticed STING monomers in the supernatant of infected VSV cultures and CD63 exocytosis to levels comparable to HSV-1(F)-infected cells. Besides VSV, we have observed exocytosis of STING monomers in ΔICP34.5 virus-infected cells. This is an HSV-1 mutant that cannot counteract autophagy, and exosomal cargo largely represents these extensive autophagolysosomal processes (37). In this case, STING enters the autophagolysosome pathway following its activation, and this is a mechanism that negatively regulates its activity. Thus, the pathway of STING exocytosis in VSV-infected cells currently remains unknown.

STING trafficking after its activation in the ER is not well described. STING resides as a dimer in the ER, and binding of 2′3′-cGAMP triggers a conformational change that facilitates STING oligomerization and exposes a cytoplasmic tail, on which TBK1 can bind (16, 38). It seems that 2′3′-cGAMP binding triggers ER-exit of STING and binding of TBK1 occurs at a post-ER location (4). Once TBK1 binds, it can phosphorylate the cytoplasmic tail of STING, which will result in the recruitment of IRF3. Activated STING can trigger type I IFN expression after its palmitoylation at the trans-Golgi network that facilitates STING oligomerization (21). STING always exists in a dimerized form; however, following HSV-1 infection, these dimers become detectable under denaturing conditions. These stable dimers were preferentially exocytosed during HSV-1 infection. One reason for easier detection of STING dimers during HSV-1 infection could be an increase in STING protein levels following its activation. However, the most likely scenario is that dimers detected upon HSV-1 infection have qualitative differences compared with STING dimers detected in a resting state that render them more stable under denaturing conditions. Notably, we did not detect phosphorylated STING (Ser-366) in EVs released by HSV-1(F) infected, but this is not surprising as p-STING (Ser-366) has not been detected in HEL cells where HSV-1(F) efficiently counteracts type I IFN responses. Also, we determined that IFNAR1 signaling was not required for STING exocytosis and blockage of IFNAR1 receptor in EV recipient cells did not significantly alter the ability of the EVs to restrict HSV-1 infection.

We have shown that CD63 colocalizes with STING in globular structures in the cytoplasm of HSV-1-infected cells, which are likely MVBs that support the packaging of STING in CD63 + exosomes. CD63 is heavily glycosylated and palmitoylated in the Golgi (39). These posttranslational modifications augment the sorting function of CD63, which is mediated by cargo binding to the large extracellular loop of CD63 or through lateral interactions with other membrane proteins (40). STING palmitoylation at the Golgi appears to facilitate its sorting into CD63 + exosomes, perhaps through lateral interactions with CD63. Inhibition of STING palmitoylation using H-151 abrogated STING exocytosis. H-151 prevents STING palmitoylation by binding to C91, and this is expected to prevent STING activation. While H-151 was anticipated to increase HSV-1 virus yields, we noticed an inhibitory effect. It is likely that the H151-bound STING caused the activation of host responses that the virus could not evade. In support of this, we noticed an increase in stable STING dimers in infected cells in the presence of H-151. Most likely, Η-151 binding increased the stability of these dimers, perhaps by preventing their degradation following ER-exit. Alternatively, compensatory mechanisms could have caused STING upregulation due to its decreased activity. We also noticed that the C88/91S substitutions reduced but did not abrogate STING exocytosis. This suggests that palmitoylation is required for optimal sorting of STING into endosomal compartments and exocytosis during HSV-1 infection and perhaps other STING modifications have a contributing role.

Another observation was that STIM1 had a contributing role in STING exocytosis during HSV-1 infection. It appears that spontaneous exit of STING from the ER in STIM1 KD cells prevented HSV-1-induced STING exocytosis. These data also imply that the virus-induced exocytosis of STING probably initiates in the ER. A decrease in ER calcium concentration that we and others have reported during HSV-1 infection could trigger the release of STIM1-bound STING from the ER (41, 42). Since 2′3′-cGAMP and the genome of the replication-defective virus ΔICP8 were not sufficient to trigger STING exocytosis, it is possible that a viral protein that traffics through the ER and Golgi alters the trafficking of STING. Multiple HSV proteins are palmitoylated during infection, and the trafficking of these viral proteins through the ER to the Golgi could trigger the clustering of STING into CD63-enriched microdomains that are released in exosomes (43). Differences in viral proteins of different herpesviruses could explain the differential trafficking of STING in EVs. For example, one of the highest palmitoylated HSV-1 proteins is glycoprotein G, but it is not palmitoylated during HSV-2 infection (43). Such a difference could be a factor affecting the virus-dependent STING exocytosis that we observed. Our data also suggest that virus post-replication events trigger STING exocytosis. Perhaps, the critical viral factor(s) involved in STING translocation to CD63 + microdomains is expressed following virus replication. Alternatively, virion envelopment and virus egress may trigger the formation of CD63-enriched microdomains in the TGN and endosomes where STING localizes. Nevertheless, the differential effect of EVs produced by HSV-1(F)- and HSV-2(G)-infected cells that differ in the amounts of exosomal STING and CD63 enhanced our previous findings showing a STING-dependent restricting effect of CD63 + EVs.

STING exocytosis during herpesviruses infection and its possible implication in restricting virus spread for the benefit of the virus has been intriguing. Recently, human rhinoviruses (HRVs) were proposed to utilize STING for the formation of virus replication compartments (44, 45). STING was proposed to contribute to the assembly and exchange of different lipid microenvironments leading to the generation of organelles essential for HRV propagation. Thus, while the STING pathway has a central role in combating pathogens, pathogens have evolved strategies to either combat or subjugate STING for their benefit.

Overall, we have elucidated the pathway of STING exocytosis during HSV-1 infection. HSV-1 triggers the trafficking of STING through the Golgi into CD63 + vesicles that are released as CD63 + EVs (Fig. 10). This is a virus-dependent process and reflects the differential effect of viruses on EV biogenesis pathways. Our data indicate that this EV biogenesis pathway could determine HSV-1 dissemination and persistence.

## MATERIALS AND METHODS

### Cell lines, viruses, and chemicals

HEL cells (immortalized human embryonic lung fibroblasts, human telomerase reverse transcriptase transformed) were cultured in Dulbecco’s modified Eagle’s medium supplemented with 10% fetal bovine serum (FBS). Vero, HEp-2, and HEK-293 cells (ATCC) were cultured according to the manufacturer’s instructions. HEK-293 STIM DKO were a gift by Dr. Gill D (Pennsylvania State University) and were maintained in DMEM + 10% FBS. HSV-1(F) and HSV-2(G) are limited-passage isolates that have been described before (46). Human herpesvirus 5 AD-169 (HCMV) (ATCC) was cultured according to the manufacturer’s instructions. VZV-32 was cultured as described previously (47). The ΔICP8 virus (d301), a kind gift from Dr. David M. Knipe (Harvard Medical School, Boston, MA), has an internal in-frame deletion in the ICP8 open reading frame (ORF) and was grown in V5-29, a Vero-derived cell line expressing the ICP8 gene (48). The ΔUL18, ΔUL36, ΔUL37 viruses, and the respective Vero cells lines that support the growth of these viruses were kindly provided by Dr. Prashant Desai (John Hopkins University, Baltimore, MD) (49–51). The 2′3′-cGAMP was purchased from Sigma (SML1229) and used at 3 µM. Η151 was purchased from Sigma (SML2437).

### Development of stable cell lines

The pLKO.1 plasmid-expressing Flag-STING was developed by inserting the Flag-STING ORF into the BamHI/SalI/Klenow site of pLKO.1 GFP CMV Puro (658-5; Addgene). Site-directed mutagenesis for the generation of the STING K150R and C88/91S mutations was performed using the QuikChange II site-directed mutagenesis kit (Agilent). HEp-2 cell lines expressing Flag-STING, Flag-STING (K150R), or Flag-STING (C88/91S) from an integrated lentiviral vector after puromycin selection were developed as described before (21, 35). The shRNA plasmids for the depletion of CD63, IFNAR1, and TBK1 were purchased from Sigma. HEL cell lines depleted of CD63 using lentiviral vectors carrying specific shRNAs were described previously (52). The pSUPER-retro-puro-shSTIM1 for the depletion of STIM1 was a gift from Shengyu Yang (Addgene plasmid # 89816) (53).

### Immunoblot analysis

Cells were solubilized in triple detergent buffer (50 mM Tris-HCl [pH 8], 150 mM NaCl, 0.1% sodium dodecyl sulfate, 1% Nonidet P-40, 0.5% sodium deoxycholate, 100 mg/mL of phenylmethylsulfonyl fluoride) supplemented with phosphatase inhibitors (10 mM NaF, 10 mM b-glycerophosphate, 0.1 mM sodium vanadate) and protease inhibitor cocktail (Sigma) and briefly sonicated. Protein concentration was determined with the Bradford method (Bio-Rad Laboratories). The mouse monoclonal antibodies to ICP8, CD63, VP16, gD, CIN85 (Santa Cruz), β-actin, Flag epitope (Sigma), ARF6 (Thermo Fisher Scientific), STING (R&D systems), Us11 (kindly provided by Dr. Roizman-University of Chicago), and STIM1 (Santa Cruz) were used in a 1:1,000 dilution. The rabbit polyclonal phospho-STING (Ser-366), TBK1, and phospho-TBK1 (Ser-172) antibodies (Cell Signaling Technology) were used in a 1:500 dilution. The anifrolumab (Invitrogen) was used at 1 µg/mL. Proteins were visualized with 5-bromo-4-chloro-3 indolylphosphate-nitroblue tetrazolium) or with ECL western blotting detection reagents (Amersham Biosciences).

### Immunofluorescence analysis

The procedures were described elsewhere (54). Briefly, the transfected/infected cells were fixed in 4% paraformaldehyde, permeabilized, blocked with phosphate-buffered saline (PBS)–TBH solution consisting of 0.1% Triton X-100 in PBS, 10% horse serum, and 1% BSA, and reacted with primary antibodies diluted in PBS–TBH. The Flag mouse monoclonal antibody (Sigma) was used at a 1:500 dilution. The cultures were rinsed several times with PBS–TBH and reacted with Alexa-Fluor-594-conjugated goat anti-mouse, diluted 1:1,000 in PBS–TBH. After several rinses, first with PBS–TBH and then with PBS, the samples were mounted and examined with a Zeiss confocal microscope equipped with software provided by Zeiss.

### Extracellular vesicle purification

Isolation of EVs from infected or uninfected cells was done as previously described with minor modifications. Briefly, HEL cells were infected with HSV-1(F), its mutant derivatives, or HSV-2(G) (0.1 PFU/cell), and EVs were isolated at 48 h post-infection. Infections with VZV were done as described previously (47), and EVs were isolated when cytopathic effects (CPE) were similar to CPE at 48 h post-infection with HSV-1(F). HCMV AD169 infections were done as described previously, and EVs were isolated when CPE was similar to CPE at 48 h post-infection with HSV-1(F) (55). The supernatant was collected, centrifuged at 500 × *g* for 5  min and at 2,000 × *g* for 20  min, and filtered through a 0.45-μm-pore-size filter. Then, the supernatant was concentrated (molecular mass cutoff, 100 kDa; Centricon Plus 70) according to the manufacturer’s instructions (Millipore). The supernatant was loaded on top of an iodixanol-sucrose gradient that ranged from 6% to 18% with a 2% or 6% increment. The 60% iodixanol was diluted in 10 mM Tris, pH 8, and 0.25 M sucrose. Samples were centrifuged in an SW41Ti rotor for 2 h at 250,000 × *g* at 4°C in a Beckman Coulter Optima XPN-80 ultracentrifuge. For functional assays, isolated EVs were washed with PBS through 100-kDa-cutoff filters (Vivaspin) and used to expose cells for 2 h prior to infecting them with HSV-1(F) or HSV-2(G) at 0.01 PFU/cell.

### Nanoparticle tracking analysis

Nanoparticle tracking analysis (NTA) was performed using the NanoSight LM10 instrument (NanoSight, Salisbury, United Kingdom). For each sample, nine different acquisitions were obtained of 60 s each. NTA software version 2.3 was used to analyze 60 s videos of data collection to obtain the mean, median, and mode of vesicle size and concentration.

### Viral DNA or RNA quantification

Total DNA was extracted from the cells using the NucleoSpin DNA RapidLyse kit (Macherey-Nagel). To detect HSV-1 DNA, we used primer pairs targeting the area of the viral genome encoding gI. To detect HSV-2 DNA, we used primer pairs targeting the area of the viral genome encoding glycoprotein G (forward, 5′-TAC GCT CTC GTA AAT GCT TC-3′, and reverse, 5′-GCC CAC CTC TAC CCA CAA-3′) (31, 56). For normalization, we used primers targeting β-actin DNA. Both primer pairs have been described before (29, 30).

### Statistical analysis and rigor

The *P* values were calculated using a standard unpaired Student’s *t*-test with a *P* ≤ 0.05 considered significant or a two-way ANOVA with Tukey *post hoc*, as indicated in figure legends. All statistical analyses were performed using at least three biological replicates. All results presented in this manuscript have been repeated in at least three independent experiments to ensure reproducibility.
